# LDIE-FDNet: Lightweight dynamic image enhancement-enabled real-time fatigue driving detection network

**DOI:** 10.1371/journal.pone.0346055

**Published:** 2026-04-01

**Authors:** Chunyu Dong, Tinglei Zhang, Jing Liu

**Affiliations:** Xi’an Key Laboratory of Human-Machine Integration and Control Technology for Intelligent Rehabilitation, School of Computer Science, Xijing University, Xi’an, China; National University of Singapore, SINGAPORE

## Abstract

Aiming at the imbalance between accuracy and real-time of existing fatigue driving detection models, and the accuracy is lower in low illumination, an LDIE-FDNet (Lightweight Dynamic Image Enhancement-Enabled Real-time Fatigue Driving Detection Network) is designed. Enhance the image by MSR-LIENET (Multi-Scale Retinex-Based Low-Light Image Enhancement Network); Through GSConv_C3k2 module, lightweight design, efficient capture of remote context information, reduction of parameters and calculation, and variable convolution kernel design and feature segmentation and mosaic are adopted to enhance feature extraction ability; Through DHFAR-Net (Dynamic Hierarchical Feature Aggregation and Reconstruction Network), combined with DySample and SDI (Semantic and Detail Infusion), the semantic information and detail information are enhanced. Through multi-level feature fusion, the model can better capture the information of various targets, reduce the situation of missing detection and false detection, thus improving the overall detection effect, and does not need high-resolution boot features as input. It has lower reasoning delay, memory occupation, floating-point operation times, and parameter number; Through the PIoU (Powerfull-IoU) loss function, IoU is calculated pixel by pixel, which can better optimize the positioning of rotating rectangular frames and deal with high aspect ratio targets, reduce the overlap of background areas and improve the detection effect. Finally, a fatigue driving detection model including maximum closing time (MCT) and maximum yawn duration (MYD) is proposed. Experiments show that mAP increases by 0.6% to 99.2, Params reduce by 24%, GFLOPs increase by 14.3% and FPS increases by 23.1% on the YawDD data set. On the DMS data set, mAP increased by 0.7% to 92.9, Params reduce by 24%, GFLOPs increased by 14.3%, and FPS increased by 20.5%. The proposed method enhances both accuracy and efficiency in fatigue detection while effectively balancing precision with real-time performance.

## Introduction

Driven by the rapid expansion of the global economy, the number of motor vehicles has surged in recent years. This increase, while improving transport convenience, has unfortunately been paralleled by a rise in traffic accidents, resulting in considerable property losses, casualties, and substantial societal safety risks. Investigation and research found that traffic accidents caused by abnormal driving behavior [1] can account for 14% ~33% of major accidents. For example, drivers’ behaviors such as answering and calling mobile phones, smoking, not wearing seat belts, and driving fatigue will seriously interfere with drivers’ attention, so that they can’t focus on observing the road conditions ahead and around them. Emergencies often overwhelm drivers, preventing timely and appropriate responses and leading to accidents with life and property losses. Abnormal driving behavior further impairs cognitive function and safety-task performance, elevating crash risk. Therefore, developing accurate, real-time algorithms for detecting driver anomalies is critical. Such systems enable real-time identification of impaired driving states, timely alerts upon anomaly detection, and ultimately enhance driving safety by preventing behavior-induced accidents. This reduces accident rates and improves road safety, offering substantial societal benefits and practical value.

Current methods for detecting abnormal driving behavior primarily fall into four categories: driver physiological characteristics-based, vehicle motion characteristics-based, driver facial features-based, and multi-feature fusion-based approaches.

### Physiological feature-based detection

Physiological feature-based fatigue detection utilizes driver biometric data to identify fatigue. This approach monitors indicators such as heart rate, eye movement, and EEG to indirectly assess the driver’s state. Feng et al. [2] extracted multiple features from Electrocardiogram and respiratory activity, and then used a fuzzy support vector machine to classify and identify different features, to predict the transition of the driver state from non-fatigue to fatigue. Zhang et al. [3] use eye-tracking technology to extract horizontal and vertical electrooculograms from forehead electrooculograms, and then calculate the ratio of eye closing time through these data, so as to evaluate the fatigue degree of drivers. Borghini et al. [4] introduced an EEG fatigue index based on alpha brain waves in this study. The researchers extracted data from the EEG of participants in monotonous driving environments and concluded that with the increase in fatigue, the frequency of alpha brain waves also increased. Amidei et al. [5] detected drowsiness of drivers by analyzing physiological signals of skin conductance. The researchers collected data through a comfortable wrist-worn device and then tested it with three integrated algorithms. The results showed that random forest had the best effect with an accuracy rate of 84.1%.

### Vehicle motion-based detection

Fatigue detection based on vehicle dynamics analyzes sensor-collected driving data to determine the driver’s state. Based on vehicle driving characteristics, mainly include steering wheel grip strength, vehicle steering angle, vehicle speed, and so on. By recording and analyzing the changing characteristics of relevant data, researchers can indirectly detect the fatigue state of drivers. Li et al. [6] proposed a four-layer recurrent neural network model based on a steering wheel angle sequence, which combines the characteristics of LSTM (Long Short-Term Memory) and RNN (Recurrent Neural Network). The model can capture the nonlinear features of the steering wheel angle series and establish a strong correlation with fatigue degree, thus obtaining the best driver fatigue feature representation method. Li et al. [7] proposed a fatigue detection system based on SWA (Stochastic Weight Averaging), which extracts and calculates ApEn (Approximate Entropy) features from steering wheel data and then uses ApEn to calculate APLA to estimate fatigue state. The accuracy of this method in fatigue state detection is 78.01%, among which 29.35% are awake and 15.15% are sleepy. Li et al. [8] developed a fatigue detection system using a fuzzy recurrent neural network (FRNN). Steering angle sensor data was collected and processed by the FRNN model, achieving an average recognition rate of 87.30%. MacDonald et al. [9] used steering wheel angle and random forest algorithm to detect lane departure, and they used the data set of the Iowa National Advanced Driving Simulator. Experimental results show that their model is more accurate than PERCLOS (Percentage of Eye Closure), and fatigue can be detected 6 seconds in advance.

### Driver facial feature-based detection

The facial feature-based approach detects driver fatigue by analyzing expressions, eye movements, and head posture. This method typically employs computer vision, image processing, and machine learning algorithms to infer the driver’s fatigue state. Xu et al. [10] employed an eye tracker to record fixation duration and pupil area measurements. Applying the Fuzzy K-Nearest Neighbors (FKNN) classification algorithm, they analyzed variations in drivers’ gaze stability and pupil dilation. Their results demonstrate that mean fixation duration combined with pupil area serves as an effective indicator for fatigue detection. Knapič et al. [11] developed a fatigue identification approach via thermal imaging-based yawn detection. This technique initially performs facial alignment through eye corner detection, subsequently utilizes long-range infrared imaging to capture yawning events, and ultimately determines driver fatigue status by quantifying yawn characteristics. Sigari et al. [12] classified fatigue levels as low, normal, and high. They used the percentage of closed eyes and the change of normal eyelid distance to detect driver fatigue. Haar-like feature detectors and fuzzy expert systems are used to detect faces in images.

### Multi-feature fusion-based driver fatigue detection

All of the above methods are based on single-mode abnormal driving behavior detection methods. Single-mode methods usually only rely on one data source or sensor, such as images, sounds, or physiological signals, which limits their applicability in different situations. Fatigue state has various expressions, and it is difficult to capture all relevant information in a single mode. External environmental conditions, such as light, noise, and vibration, may interfere with single-mode detection methods, resulting in performance degradation. The integrated multi-feature fusion approach effectively addresses these limitations, enhancing model accuracy while improving stability and generalization capability. Zhuang [13] and others put forward a method of eye fatigue detection based on pupil and iris segmentation. This method uses a segmentation network to extract pupil and iris features of eye images and uses a decision network to evaluate the degree of eye-opening and closing. Finally, the driver’s state is predicted by calculating the PERCLOS value. Zhao [14] and others put forward an intelligent detection model combining face detection, face key point location, and fatigue decision. First, face detection is carried out by the YOLOv4-tiny network, then the face key point location is carried out by the PFLD network, and finally, the fatigue state is judged by the decision network. Zhang et al. [15] developed a fatigue detection system utilizing facial recognition with an enhanced YOLO algorithm. Their framework incorporates the YOLOv5 architecture, introducing Label Smoothing in the prediction layer. By implementing a central loss function, the approach minimizes intra-class feature variance while preserving inter-class separability. Xiang et al. [16] designed a 3D CNN-based fatigue detection system integrated with a channel attention mechanism. This integration optimizes feature weighting through dedicated attention modules, significantly enhancing detection performance. Zheng et al. [17] proposed a deep learning-based MAX-MIN algorithm for driver fatigue detection. The method mitigates environmental interference impacts and computes Eye Aspect Ratio (EAR) and Mouth Aspect Ratio (MAR) through comparative image analysis. Fang et al. [18] proposed a time-varying driver characteristic-aware shared control framework that dynamically adapts to evolving driver states, enhancing safety and comfort in intelligent vehicles through real-time physiological and behavioral modeling. Building upon this, Fang et al. [19] introduced a trust-based authority allocation strategy for steering control, where human-machine mutual trust levels directly govern control authority distribution, optimizing cooperative driving performance under varying trust dynamics. Selvan et al. [20] developed a deep learning framework using sensor-fused IoT for real-time fetal health classification, achieving 96.7% accuracy by integrating multimodal physiological data (ECG, uterine contractions, fetal heart rate) via convolutional neural networks.

Traditional feature-based detection mode is mostly carried out by feature extraction and shallow network training model. Although this mode has fast detection speed, the detection accuracy is unstable. The detection mode based on deep learning is mostly a training network characterized by the deep network. Because of the many layers and parameters of the network, although the precision of precision measurement is high, it also leads to the decline of detection speed and also increases the use of computing resources and memory. At the same time, due to the difference in the driver’s sitting posture and position, the scale difference between target features and the angle of extracted target features will be tilted, which will affect the detection accuracy. Because fatigue driving detection requires high real-time detection accuracy, it is not practical to use a traditional deep learning network to detect abnormal driving behavior.

Therefore, this paper designs LDIE-FDNet (Lightweight Dynamic Image Enhancement-Enabled Real-time Fatigue Driving Detection Network), which includes MSR-LIENET (Multi-Scale Retinex-Based Low-Light Image Enhancement Network) [21], Backbone, Neck and Head. These strategies effectively address key challenges in complex in-vehicle environments: low detection accuracy, slow processing speed, and excessive computational resource/memory consumption during driver image analysis. In the Backbone part, a multi-scale feature pyramid is constructed to extract features, and the GSConv_C3k2 module is designed in the second, third, fourth, and fifth layers of the pyramid, which can realize lightweight design, effectively capturing context information and reducing parameters and computation, thus improving detection accuracy and speed. To reduce the impact of complex backgrounds and different target scales, The Neck module performs multi-scale feature enhancement through hierarchical extraction and integration and proposes DHFAR-Net (Dynamic Hierarchical Feature Aggregation and Reconstruction Network), which effectively enhances semantic information and detail information, does not need high-resolution guiding features as input, and has lower reasoning delay, memory occupation, floating-point operation times and parameter number. The head module projects extracted features onto the output space for network prediction generation. To enhance bounding box similarity assessment, the predicted and ground truth annotations are rigorously compared, this paper uses PIoU (Powerful-IoU), which can better adapt to high aspect ratio targets (smoking, safety belts, eyes, etc.). By calculating the IoU pixel by pixel, PIOU loss can better deal with this kind of target, reduce the overlap of background areas, and improve the detection effect. Our proposed LDIE-FDNet has the following advantages:

1. MSR-LIENET greatly improves the detection accuracy of low-illumination images.
2. The GSConv_C3k2 module is proposed, which combines the C3k2 and GSConv [22] modules. It is lightweight, captures remote context information efficiently, reduces the amount of parameters and calculation, and adopts variable convolution kernel design feature segmentation, and mosaic to enhance the feature extraction ability.
3. DHFAR-Net is proposed, which combines DySample [23] and SDI (Semantic and Detail Infusion) [24] to enhance semantic information and detail information. Through multi-level feature fusion, the model can better capture the information of various targets, reduce missed detection and false detection, and thus improve the overall detection effect. And does not require high-resolution boot features as input, with lower inference latency, memory footprint, floating-point operations, and the number of parameters.
4. PIoU [25] loss function is introduced. PIOU loss is calculated pixel by pixel, which can better optimize the positioning of a rotating rectangular frame and deal with high aspect ratio targets, reduce the overlap of background areas, and improve the detection effect. Moreover, the PIoU loss function is continuous and differentiable, which means that it can be effectively optimized by gradient descent and other methods in the training process, without the problem of gradient disappearance or explosion, thus ensuring the stability and effect of training.
5. MCT(Maximum closing time) and MYD(maximum yawn duration) are proposed to determine the fatigue and the dangerous driving behavior of eyes closed and yawning. The results are as follows: MCT and MYD are used to determine the fatigue dangerous driving behavior of eyes closed and yawning fatigue behavior of eyes closed and yawning fatigue behavior of eyes closed and yawning fatigue.

### Contributions and novelty

Contributions and Novelty. This work makes the following contributions:

1. Task-driven low-light-to-detection coupling: We integrate MSR-LIENET as a pre-enhancement stage for in-cabin low illumination and evaluate its impact on downstream detection performance.
2. Backbone lightweight redesign (GSConv_C3k2 placement): We replace standard convolutions inside the C3k2-based pyramid (layers 2–5) with GSConv_C3k2 blocks to reduce redundant computation while preserving cross-channel feature interaction for subtle facial cues.
3. Neck redesign (DHFAR-Net): We propose a Dynamic Hierarchical Feature Aggregation and Reconstruction Network that combines DySample-based dynamic upsampling and SDI-based semantic/detail infusion to improve multi-scale fusion under occlusion and pose variations.
4. Regression optimization for elongated targets: We introduce PIoU loss to better localize high-aspect-ratio behaviors (e.g., closed eyes, cigarette, seatbelt) that are common in driver monitoring.
5. System-level fatigue decision module: We further define MCT and MYD metrics based on detection outputs to convert frame-level detections into fatigue-state decisions.

## Materials and methods

### Overall architecture

End-to-end formulation. Given a video sequence ${\left\{I_{t}\right\}}_{t=1}^{T}$, we first enhance each frame using MSR-LIENET:


$${\tilde{I}}_{t}=E\left(I_{t}\right)$$ (1)

where E(·) denotes the low-light enhancement network. The detector produces behavior detections:


$$D_{t}=F_{\theta }\left({\tilde{I}}_{t}\right)$$ (2)

where $F_{\theta }$ is LDIE-FDNet with parameters $\theta_{1}$ and *D*_*t*_ contains bounding boxes, class labels, and confidence scores. Finally, fatigue decision is obtained by computing MCT and MYD over frame-level detections:


$$y=G\left({\left\{D_{t}\right\}}_{t=1}^{T}\right)$$ (3)

where G is the rule-based decision function defined in Section “Fatigue evaluation.”

YOLOv11 [26], the current state-of-the-art iteration from Ultralytics, extends the lineage of its predecessors by incorporating novel enhancements that boost both computational efficiency and adaptability. This architecture integrates refined backbone and neck modules, strengthening feature representation for improved object detection precision and complex scene adaptability. C3K2 module and C2PSA module are introduced. All components are optimized comprehensively, including:

1. Enhanced feature extraction: YOLOv11’s refined backbone-neck architecture enhances feature discriminability, thereby elevating detection precision and complex scene perception capabilities.
2. Optimize efficiency and speed: Fine architecture design and optimized training pipeline make YOLOv11 provide faster processing speed while maintaining high precision, and achieve the best balance between accuracy and performance.
3. Using fewer parameters to obtain higher accuracy: On the COCO data set, YOLOv11m achieves higher average accuracy (mAP), and uses 22% fewer parameters than YOLOv8m [27], thus improving computational efficiency without sacrificing accuracy.
4. Cross-environment adaptability: YOLOv11 exhibits cross-platform compatibility across heterogeneous systems—from edge computing nodes to cloud infrastructures and NVIDIA GPU-accelerated environments—ensuring deployment versatility without compromising efficacy.

**Fig 1:**
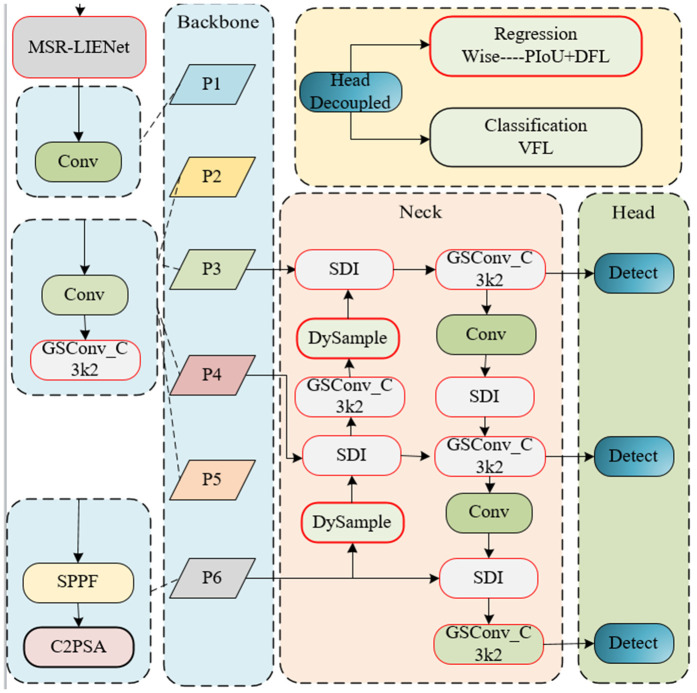
Depicts the LDIE-FDNet architecture. The Backbone establishes a six-tier feature pyramid for multi-scale extraction, while the Neck employs a dynamic hierarchical fusion pyramid for feature refinement. The Head handles localization and classification, with PIoU Loss enhancing geometric characterization of target morphology and spatial configuration.

We propose an LDIE-FDNet network based on YOLOv11n, which consists of four parts: MSR-LIENET, Backbone, Neck, and Head. As shown in Fig 1, Backbone builds a six-layer feature pyramid through a CNN network to extract information with different scales and features. Layer 1 is composed of Conv modules, layers 2, 3, 4, and 5 are composed of Conv modules and GSConv_C3k2 modules, and layer 6 is composed of SPPF and C2PSA modules. It realizes lightweight design, captures remote context information efficiently, reduces parameters and computation, and adopts variable convolution kernel design, feature segmentation, and mosaic to enhance feature extraction ability. After that, the feature map is further extracted and integrated into the Neck layer. DHFAR-Net is proposed in this layer, which combines DySample and SDI to enhance semantic information and detail information. Through multi-level feature fusion, the model can better capture the information of various targets, reduce missed detection and false detection, and thus improve the overall detection effect. And does not require high-resolution boot features as input, with lower inference latency, memory footprint, floating-point operations, and a number of parameters. The Head layer processes Neck-derived features to execute detection operations, predicting target locations and categories. For regression optimization, the PIoU loss function enhances the geometric characterization of target position and morphology. The network subsequently generates probabilistic outputs comprising bounding box coordinates and categorical probabilities.

Table 1 clarify information flow and computational behavior

**Table 1:** Information flow and computational behavior.

Challenge in driver monitoring	Baseline limitation (YOLOv11n)	Our modification	Expected effect
Low cabin illumination	features degraded / low contrast	contrast MSR-LIENET enhancement before detection	improve visibility, stabilize features
Subtle facial cues (eyes/mouth)	channel interaction limited with DWConv	GSConv_C3k2 in pyramid layers 2–5	preserve cross-channel cues with fewer params
Multi-scale + occlusion	fixed upsampling + simple concat	DHFAR-Net = DySample + SDI replacing upsample/concat	better fusion of semantics + details
Elongated targets (eyes/cigarette/seatbelt)	CIoU may regress poorly	PIoU loss for box regression	mproved localization
Fatigue state needs temporal logic	detection only (frame-level)	MCT/MYD decision rules	turn detections into fatigue state

### MSR-LIENET

Developed by Land in the 1970s-1980s, Retinex theory explains human color perception through retinal-cortical interactions. This neurophysiological model accounts for color constancy—the ability to perceive consistent chromatic information under varying illumination. As a foundational computer vision framework, the Retinex model integrates principles of color constancy and trichromatic theory for image processing applications. Retinex theory decomposes image I(x,y) into illumination L(x,y) and reflection R(x,y) components, representing incident light characteristics and object reflectivity respectively. This relationship is formally expressed by Eq (4).


I(x,y)=R(x,y)⊙L(x,y) (4)

Where ⊙ means multiplication by element. Empirical analysis of low-light image enhancement reveals distinct linear correlations across RGB channels when comparing dimmed and standard illumination images. Channel-specific enhancement gradients emerge due to differential spectral energy distributions between illumination conditions, resulting in varied slope coefficients per chromatic channel. Even if the low illumination image data sets generated by different exposure times, such as LOL data sets, are processed by camera image signals such as white balance and color correction, the enhancement ratio among the three RGB channels is different. Therefore, when low illumination enhancement is carried out, Eq (4) is changed to Eq (5).


$$I(x,y)=R(x,y)⊙L_{c}(x,y)$$ (5)

Among them, c∈ {R, G, B}, in the illumination enhancement module, the illumination of the three channels is enhanced respectively to avoid the generation of color shift.

**Fig 2:**
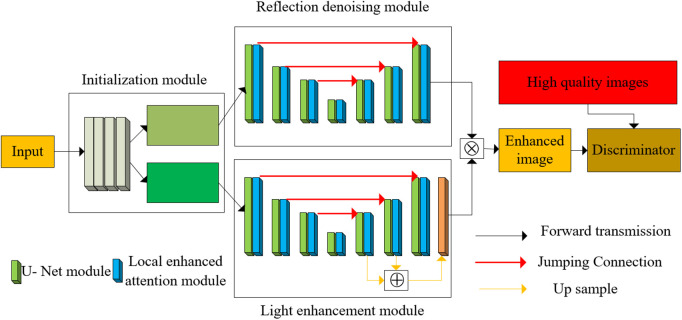
Low-light image enhancement network based on multichannel Retinex model.

Because the illumination component R and reflection component L are difficult to obtain accurately, the depth network is used to fit R and L adaptively. Therefore, based on the above optimization scheme, the Retinex decomposition and low illumination enhancement process are mapped to the depth unfolding network architecture, and a new framework for low illumination image enhancement is proposed. As illustrated in Fig 2, the multi-channel Retinex-based low-light enhancement network employs a generator-discriminator framework. The generator comprises three specialized modules: Initialization, Reflectance Denoising, and Illumination Enhancement. The initialization module decomposes input images into illumination and reflectance components with high fidelity. These outputs undergo parallel processing—reflectance denoising and illumination enhancement—followed by multiplicative recomposition to generate the final enhanced image. The discriminator judges whether the image is perceived to be of high quality through antagonistic loss by comparing it with the artificially selected high-quality picture. In this way, good results can be achieved in both signal fidelity and human perceptual quality.

### GSConv_C3k2

In real abnormal driving behavior detection, the background is complex and time-sensitive, so it is a great challenge to extract abnormal behavior features from driving images with minimum computation. As an optimized version of CSPBottleneck, the C3K2 module is designed to improve computational efficiency. C3K2 reduces the computational complexity through several layers of compact convolution structure but still maintains the ability of deep feature extraction.

C3k2 module, as a faster implementation of CSPBottleneck, improves the efficiency of data processing by adopting two parallel convolution layers. In C3K2, important parameter adjustment makes it more advantageous in processing speed, which ensures that the processing delay is controlled while maintaining accuracy, and is suitable for real-time application scenarios such as abnormal driving behavior.

SC(Standard Conversion) is often used for feature extraction. As shown in the figure, it has a strong global feature aggregation ability, but the real-time performance is limited by the number of disabled persons and computational complexity, and its computational complexity is shown in Eq (6).


$$T_{SC}=W\cdot H\cdot K^{2}\cdot C_{1}\cdot C_{2}$$ (6)

Denoting: W, H: spatial dimensions of the feature map (width × height); K: convolutional kernel size; C1, C2: channel depth of input/output feature maps. It can be seen from equation (1). The computational complexity increases linearly with the resolution (WxH) and proportionally with the product of channel numbers (C1xC2), which leads to high resource consumption for high-resolution or multi-channel scenes and limits real-time applications.

In order to reduce the computational complexity, DWConv [28] (Depthwise Separable Convolutions) has gradually attracted attention, and deep separable convolution is one of the common schemes. DWConv significantly reduces the computational complexity through the decomposition design of channel-by-channel convolution and point convolution. However, channel-by-channel convolution processes feature independently, which does not fully model the correlation between channels, resulting in incomplete feature integration. This limitation weakens the continuity of information expression, and it is insufficient in tasks such as fatigue driving detection, which needs to extract subtle features.

**Fig 3:**
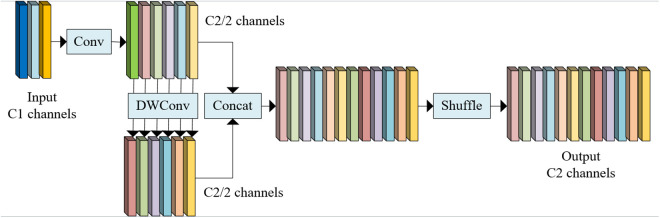
Conversion Structure Of GSConv.

Therefore, this paper introduces GSConv, a lightweight convolution module that takes into account both computational efficiency and feature integrity. As shown in Fig 3, GSConv can reduce the computational cost and enhance the ability to extract subtle features and modeling information between channels through structural optimization, which effectively makes up for the shortcomings of DWConv.

GSConv includes the following three key steps: First, the standard convolution is used to reduce the dimension of the input feature C1, and the number of channels is reduced to C2/2, which can effectively capture the basic features while reducing the computational complexity. Secondly, DWConv is used to process features independently channel by channel, and a sparsity strategy is introduced to enhance feature diversity and detail expression ability. Finally, the output of standard convolution and depth convolution are fused to generate a complete feature map with the number of channels C2, and the channel information distribution is optimized by channel shuffling operation to improve the cross-channel feature interaction ability. This design not only solves the problem of incomplete feature integration but also captures the subtle features in complex scenes more accurately, providing a solid foundation for learning complex patterns on the Internet. The computational complexity is shown in Eq (7).


$$T_{GSConv}=W\cdot H\cdot \left(k_{1}^{2}\cdot C_{1}\cdot \frac{C2}{2}+k_{2}^{2}\cdot \frac{C_{2}^{2}}{2}\right)$$ (7)

Where: k1 and k2 are convolution kernel sizes. Compared with standard convolution, GSConv reduces computational complexity and maintains excellent feature extraction ability through dimension reduction, sparse design, and channel optimization strategy.

**Fig 4:**
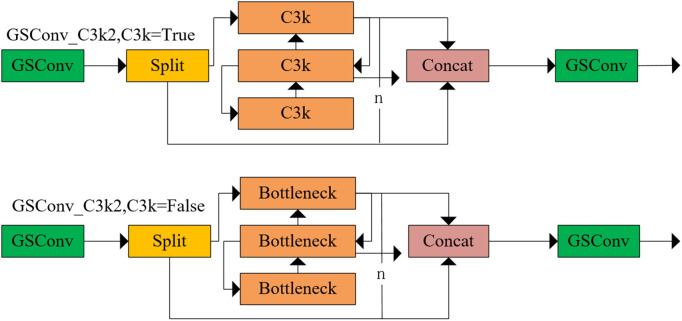
GSConv_C3k2 structure.

To optimize computational efficiency while preserving detection fidelity, GSConv is introduced into the C3K2 module, and the GSConv_C3k2 module is designed to replace the original standard convolution structure. In the feature extraction stage, GSConv_C3k2 effectively mitigates computational resource demands through dimensionality reduction, sparse design, and channel optimization, thus realizing the lightweight detection model. Fig 4 illustrates the structural configuration.

### DHFAR-Net

The Neck is the middle layer connecting the Backbone and Head. The main function of the Neck is to reduce the dimension or adjust the features of the Backbone to better adapt to the task requirements. The Neck conducts multi-scale feature aggregation and propagates fused representations to the prediction layer.

To enhance cross-scenario generalization capabilities, improve the efficiency of abnormal driving behavior detection, and improve the efficiency of detection accuracy without loss, DHFAR-Net is proposed in the Neck part, and DySample and SDI are introduced. Through the introduction of this module, the detection efficiency of the network is greatly improved, which is more conducive to the real-time detection of abnormal driving behavior.

#### DySample module.

Current up-sampling methods (interpolation and transposed convolution) use fixed sampling, failing to adapt to feature content and compromising semantic integrity. While kernel-based dynamic up-sampling improves detection, it introduces heavy computational loads, hindering real-time abnormal driving monitoring. To address this, we integrate a lightweight DySample operator into the neck network. DySample employs point sampling for dynamic up-sampling, avoiding complex convolution and sub-networks in traditional methods, thus significantly reducing parameters while preserving fine details and capturing subtle features.

DySample enhances model robustness by directing sampling points toward target regions while suppressing background interference, improving anti-interference capability. Compared to kernel-based dynamic up-sampling, it reduces parameters and computational load, enabling real-time abnormal driving detection.

**Fig 5:**
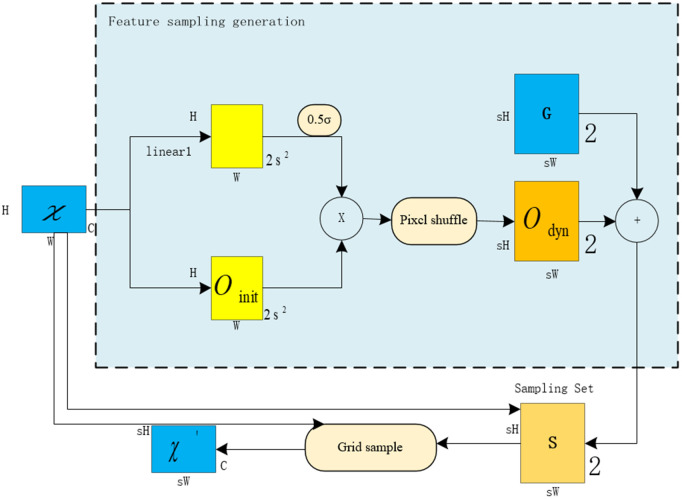
DySample structure.

The DySample structure is shown in Fig 5. First, an input feature map with a given size of C × H × W is transformed by a sample point generator into a sample set S with a size of 2 × sH × sW, where 2 of the first dimension represents X and Y coordinates. Then the input feature is re-sampled by using the gridsample function, and the up-sampled feature graph with the size of C × sH × sW is obtained. In the design of DySample, the sampling point generator is the key part to realize efficient up-sampling. In this part, the offset S is generated by combining linear layer, dynamic range factor, and pixel shuffle sub-pixel convolution. The offset vector S is then element-wise added to the original grid coordinates G, yielding the final sampling set S. Incorporating a dynamic range factor enhances offset adaptability while mitigating excessive sampling concentration.

This process can be expressed by Eq (8):


$$o=0.5sigmoid\left(linear_{1}\left(\chi \right)\cdot linear_{2}\left(\chi \right)\right)$$ (8)

The strengths of DySample are:

1. DySample is a design based on point sampling, which divides a point into multiple points to achieve clearer edges, while CARAFE [29], FADE, and SAPA [30] are dynamic up-sampling methods based on convolution kernel, which realize up-sampling by generating dynamic convolution kernel or clustering.
2. DySample does not need high-resolution boot features as input, nor does it need additional CUDA packets, while CARAFE, FADE, and SAPA methods usually need high-resolution boot features and additional subnetwork or dynamic convolution operations.
3. DySample realizes the up-sampling process by dynamic sampling to find the correct semantic clustering of each up-sampling point, while CARAFE, FADE, and SAPA usually use dynamic convolution or clustering to recombine input features.
4. DySample is more lightweight in implementation, does not need a customized CUDA package, and has lower computing resource consumption, while CARAFE, FADE, and SAPA usually need more parameters, FLOPs, GPU memory, and reasoning time.

#### SDI module.

**Fig 6:**
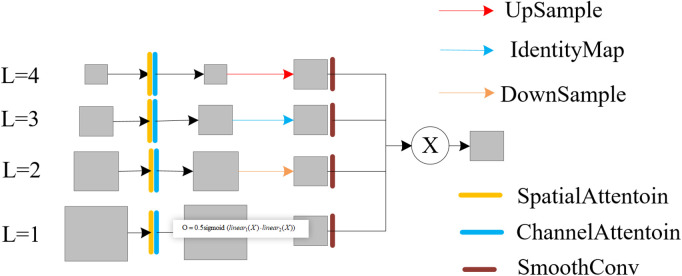
Architecture of the SDI module. For clarity, only the refinement process for the third-level features (l = 3) is depicted. SmoothConv denotes a 3x3 convolution for feature smoothing, and X represents the Hadamard product.

SDI is a module for users to replace Concat operation, which enhances semantic information and detail information in images by integrating multiple levels of feature maps generated by encoders. Its working principle is as follows: (1) Feature extraction: Firstly, the encoder generates multi-level feature maps for the input image. Feature maps processed by the SDI module encompass multi-scale information, spanning low-level detail to high-level semantic content. Within this module, spatial and channel attention mechanisms are applied to features at each scale. This dual attention facilitates the integration of local spatial cues and global channel statistics, boosting feature discriminability. Fusion of high-level (semantically rich) and low-level (fine-detail) features is performed per level, commonly via Hadamard product [31] or analogous methods. Subsequently, the enhanced features are decoded for applications including image reconstruction, segmentation, and object detection, as depicted in Fig 6.

Replacing Concat with an SDI module has the following advantages:

1. Enhances accuracy: By mitigating information loss and semantic misalignment through multi-level feature fusion, the SDI module significantly improves the precision of segmentation and detection tasks.
2. Maintain computational and memory efficiency: Although the complexity of feature fusion is increased, the SDI module still maintains computational and memory efficiency, which makes it feasible in practical applications.

### PIoU module

The CIoU loss [32] incorporates penalty terms for center point distance and aspect ratio discrepancy but fails to directly model the geometric dissimilarity between the predicted and ground truth boxes. This limitation can lead to suboptimal convergence behavior, and the penalty term in CIoU does not reflect the change in the size of the target frame. In the actual abnormal driving behavior detection process, it is found that the CIoU loss function will lead to the problem of low positioning accuracy for targets with high aspect ratios and slender inclinations. To address this limitation, this paper introduces PIoU into the loss function, which calculates the intersection-union ratio by counting the internal pixels of the image, and the loss is sensitive to the size and position of the bounding box. The PIOU loss function combines the target size adaptive penalty factor and the gradient adjustment function based on the quality of the detection box to guide the detection box to regress along the effective path, thus converging faster than the existing IOU-based loss. PIOU is specifically shown in Eq (9)-Eq (12):


$$P=\frac{\frac{dw_{1}}{w_{gt}}+\frac{dw_{2}}{w_{gt}}+\frac{dh_{1}}{h_{gt}}+\frac{dh_{2}}{h_{gt}}}{4}$$ (9)

$$f\left(x\right)=1-e^{-x^{2}}$$ (10)

PIoU=IoU−f(P),−1≤PIoU≤1 (11)

$$L_{PIoU}=1-PIoU=L_{IoU}+f\left(P\right),0\leq L_{PIoU}\leq 2$$ (12)

**Fig 7:**
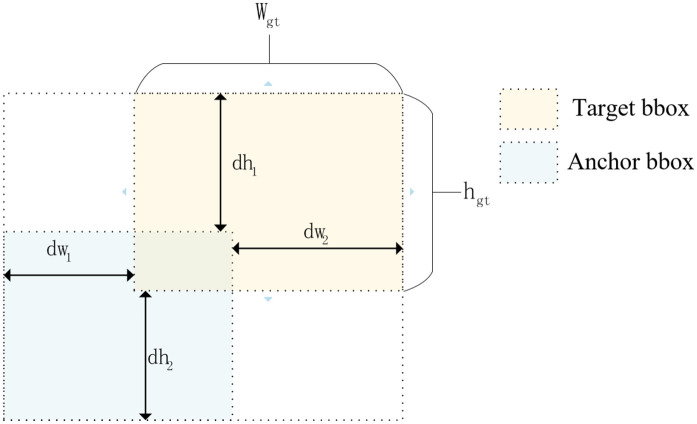
PIoU conceptual structure.

Here, P denotes a target-size-adaptive penalty factor, ensuring the loss function intrinsically adapts to object scale without inducing bounding box inflation. Variables dw1, dw2, dh1, and dh2 represent the absolute distances between the predicted box and corresponding edges of the ground truth box, while wgt and hgt denote the width and height of the ground truth box, respectively. The function f(x) dynamically modulates gradient magnitudes based on detection quality. The conceptual framework of PIoU is illustrated in Fig 7.

PIoUv2 [33], a novel loss function, integrates a focusing mechanism with a non-monotonic attention module into the PIoU framework. This formulation prioritizes medium-quality detection boxes, as detailed in Eq (13)-Eq (15).


$$q=e^{-p},q\in \left(0,1\right]$$ (13)

$$u\left(\chi \right)=3\chi \cdot e^{-x^{2}}$$ (14)

$$L_{PIoU_{V2}}=u(\lambda q)\cdot L_{PIoU}=3\cdot (\lambda q)\cdot e^{-(\lambda q{)}^{2}}\cdot L_{PIoU}$$ (15)

Eq (15), u(λq) denotes the attention function where the penalty factor p is substituted by a quality metric q. This metric q∈(0,1] quantifies detection box confidence and serves as a hyperparameter u(λq) governing the attention function’s behavior. The non-monotonic formulation f(x) enhances PIoU’s focus on medium-quality detection boxes. Requiring only a single hyperparameter, PIoUv2 significantly streamlines model tuning.

## Fatigue evaluation and fatigue driving detection model

We propose two evaluation criteria to quantify fatigue states. Fatigue state can be divided into closing eyes, yawning, smoking, using mobile phones, etc. Smoking and using mobile phones are directly judged as fatigue-dangerous driving, while MCT and MYD are needed to judge fatigue-dangerous driving for closing eyes and yawning respectively.

### MCT

In the actual driving process, drivers will close their eyes for a long time because of fatigue, which should be warned. In this paper, the index of the longest eye-closing time is proposed. When the driver starts to close his eyes, the calculation of the continuous eye-closing time is shown in Eq (16).


$$T_{e}=\left(E_{end}-E_{start}\right)*T$$ (16)

where: *T*_*e*_: Total duration of eye closure; *E*_*end*_: Frame index from eye closure onset to reopening; *E*_*start*_: Starting frame index of eye closure; T: Per-frame acquisition time interval.

This study establishes a fatigue detection criterion wherein sustained eye closure exceeding 2 seconds indicates driver fatigue. Given the dataset’s average video frame rate of 20 fps (corresponding to 50ms per frame), consecutive eye closure spanning > 40 frames triggers fatigue state detection.

### MYD

Apart from eye closure, yawning behavior also manifests when a driver is in a fatigued state. This paper proposes the longest yawning duration indicator, which calculates the duration of sustained mouth opening as shown in Eq (17).


$$T_{m}=\left(M_{end}-M_{start}\right)*T$$ (17)

Where: *T*_*m*_ represents the duration of sustained mouth opening; *M*_*end*_ denotes the frame sequence number when the mouth closes after sustained opening; *M*_*start*_ denotes the frame sequence number when mouth opening begins; T represents the time interval for acquiring each frame.

This paper establishes that a driver is in a fatigued state when the Longest Yawning Duration indicator exceeds 3 seconds. That is, if the mouth remains open for more than 60 consecutive frames, the driver is determined to be fatigued.

### Threshold justification

We analyze the distributions of eye-closure duration *T*_*e*_ and yawning duration *T*_*m*_ in the evaluation set. Fatigue-related events exhibit substantially longer durations than normal blinks/mouth movements, and the separation becomes stable around the 2 s (eye closure) and 3 s (yawning) thresholds. These thresholds are also consistent with commonly adopted fatigue criteria reported in prior driver monitoring studies [34,35]. Therefore, we adopt MCT > 2s and MYD > 3s as fatigue triggers in our system-level evaluation.

### Driver fatigue recognition system

**Fig 8:**
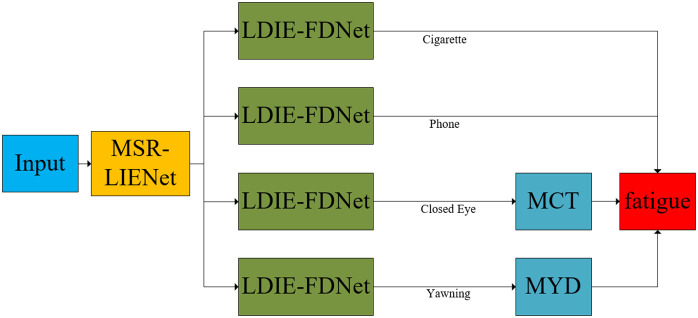
Driver fatigue recognition system.

In conclusion, this work establishes a driver drowsiness detection framework, with its architectural configuration detailed in Fig 8.

The proposed driver fatigue detection algorithm operates as follows:

1. Frame Extraction: Input videos are segmented into sequential frames.
2. Image Enhancement: Frame images undergo contrast enhancement via MSR-LIENET.
3. Fatigue Detection: Enhanced images are processed by LDIE-FDNET network.
4. State Assessment: MYD and MCT metrics determine driver fatigue status.

## Experiment

### Datasets

We evaluate the performance of the proposed LDIE-FDNet network on YawDD [36] facial fatigue driving detection data set and DMS (Driver Monitoring System) [37] fatigue driving data set. YawDD data set is a video data set obtained by collecting and labeling the facial features of drivers, including videos of different individuals of different genders closing their eyes and yawning while driving. Table 2 lists the details of the dataset.

**Table 2:** Detailed introduction of YawDD data set.

*Target Classes*	Target Numbers
*Closed Eye*	5276
*Yawning*	5569

DMS data set is a data set specially designed for a driver monitoring system, which aims to train the model to accurately identify drivers’ behaviors such as opening and closing eyes, smoking, using mobile phones, and wearing seat belts. These tests are of great significance for evaluating drivers’ concentration and ensuring driving safety. The class distribution of the DMS dataset is summarized in Table 3.

**Table 3:** Details of DMS Datasets.

*Target Classes*	Target Numbers
*Open Eye*	11153
*Closed Eye*	1596
*Cigarette*	3907
*Phone*	1882
*Seatbelt*	4149

#### Dataset splits and unseen-driver protocol.

For the YawDD dataset, we split it into training/validation/test sets with a ratio of 70:10:20. To evaluate generalization to new subjects, we adopt a subject-independent split strategy, where the identities in the test set do not appear in the training or validation sets. Specifically, 7393 drivers are used for training, 1056 for validation, and 2112 unseen drivers for testing.

For the DMS dataset, we follow the same protocol and ensure no identity leakage across different splits. The exact split lists are provided in our public repository to ensure reproducibility.

### Performance metrics and experimental configuration

#### Evaluation indicators.

To validate the proposed network’s efficacy for classroom behavior recognition, we employ three performance metrics: Precision(P), Recall(R), and mean Average Precision(mAP). These metrics are formally defined as follows:


$$Precision=\frac{TP}{TP+FP}$$ (18)

$$Recall=\frac{TP}{TP+FN}$$ (19)

Where: TP: True positives (correctly detected classroom behaviors); FP: False positives (detections incorrectly classified as target behaviors); FN: False negatives (undetected target behaviors).


$$mAP=\frac{\sum_{n=1}^{N}AP_{n}}{N}$$ (20)

Let: N: Total number of categories; K: Detection count; AP: Per-category average precision.

The standard mAP is computed at two IoU thresholds: mAP50: IoU threshold fixed at 0.5; mAP50-95: Average mAP over IoU thresholds ranging from 0.5 to 0.95 (inclusive, with 0.05 increments).

This protocol holistically evaluates multi-category detection performance by:

1. Overcoming single-category assessment limitations
2. Providing comprehensive algorithm benchmarking
3. Quantifying model stability across categories
4. Enabling intuitive cross-study comparisons

Consequently, mAP has emerged as the de facto evaluation standard for object detection tasks.

#### Experimental environment.

Implementation details of YOLO: We adopt the Ultralytics implementation of YOLOv11n (Ultralytics version: v8.3.15, commit: 22ebd44f62791cfd4de2a24de15ce05a13c1447). For a fair comparison, YOLOv8n and YOLOv5n were trained with the same input resolution, dataset splits, and training schedule as YOLOv11n.

The training configuration was set as follows:

1. Total epochs: 500;
2. Batch size: 4;
3. Data loading workers: 8;
4. Input resolution: 640×640 pixels.

Optimization employed Stochastic Gradient Descent (SGD) with: Weight decay: $5*10^{-4}$ (L2 regularization); Nesterov momentum: 0.937. Early stopping was implemented to automatically terminate training when validation loss plateaued, indicating model convergence.

The experimental environment of the model is the Ubuntu 20.04 operating system, with PyTorch2.0. 1, Python4.9.19 and CUDA12.2.79 installed, CPU Intel (R) Core (TM) i7-9700KF CPU@4.60GHz, and GPU NVIDIA GeForce GTX 1050 Ti. For an input image with a size of 640x640 pixels and three color channels, the proposed method includes 381 layers, including 1.92 million parameters, 5.4 GFLOPs, 300poch training model, and 4.27 m model size.

#### Statistical reporting.

To reduce randomness, we repeated training with three random seeds (0, 1, 2) for key models and report the mean ± standard deviation of mAP and FPS. We further perform a paired t-test between the baseline and LDIE-FDNet results to verify whether improvements are statistically significant (p < 0.05).

### Ablation test

#### Overall experiment of algorithm.

To further evaluate the effectiveness of each module, ablation studies were conducted on the YawDD and DMS datasets. Using YOLOv11n as the baseline, Table 4 presents the results for the YawDD dataset.

**Table 4:** Ablation experiment of YawDD dataset.

MSR-LIENET	GSConv_C3k2	DySample	SDI	PIoU	mAP50(%)	Params(M)	GFLOPs	PS
×	×	×	×	×	98.6	2.5	6.3	39
√	×	×	×	×	99.7	2.5	6.3	32
×	√	×	×	×	98.5	2.3	5.9	41
×	×	√	×	×	98.4	2.3	6.1	40
×	×	×	√	×	98.7	2.2	6.0	41
×	×	×	×	√	98.7	2.4	6.2	44
√	√	×	×	×	99.4	2.3	5.9	33
√	×	√	×	×	99.6	2.3	6.1	34
√	×	×	√	×	99.5	2.2	6.0	33
√	×	×	×	√	99.5	2.5	6.3	34
×	√	√	×	×	98.4	2.2	5.8	42
×	√	×	√	×	98.4	2.3	6.0	41
×	√	×	×	√	98.6	2.1	5.9	43
×	×	√	√	×	98.5	2.2	6.2	44
×	×	√	×	√	98.2	2.3	6.1	42
×	×	×	√	√	98.4	2.1	6.1	42
√	√	√	×	×	99.4	2.2	5.8	36
√	√	×	√	×	99.3	2.3	6.0	36
√	√	×	×	√	99.5	2.1	5.9	35
√	×	√	√	×	99.5	2.2	6.2	7
√	×	√	×	√	99.4	2.3	6.1	36
√	×	×	√	√	99.5	2.1	6.1	34
×	√	√	√	×	98.1	2.1	5.7	49
×	√	√	×	√	98.2	2.2	5.8	49
×	√	×	√	√	98.1	2.1	5.7	50
×	×	√	√	√	98.1	2.0	5.6	9
√	√	√	√	×	99.3	2.1	5.7	46
√	√	√	×	√	99.4	2.2	5.8	47
√	√	×	√	√	99.3	2.1	5.7	46
√	×	√	√	√	99.3	2.0	5.6	47
×	√	√	√	√	98.0	1.9	5.4	52
√	√	√	√	√	99.2	1.9	5.4	48

The baseline (YOLOv11n) achieved an impressive mAP50 of 98.6%. This high performance stems from its backbone architecture, centered on the C3K2 module—an evolution of the CSP bottleneck. C3K2 employs smaller 3x3 kernels for efficient computation while effectively capturing basic image features. It optimizes information flow by splitting the feature map, processing the splits with computationally efficient 3x3 convolutions, and merging them later. Compared to YOLOv8’s C2f module, C3K2 achieves superior feature representation with fewer parameters. The architecture retains the SPFF module for multi-scale feature aggregation, enhancing detection capability across object sizes, particularly small objects. A key innovation is the C2PSA module, which incorporates an attention mechanism to model spatial dependencies, directing focus towards critical regions like small or partially occluded objects. While effective in natural scenes, applying this model to fatigue driving detection poses challenges due to typically suboptimal lighting conditions and stringent real-time requirements, indicating limitations for this specific application.

Consequently, we proposed the integration of MSR-LIENET for low-light image enhancement, leading to a 1.1% improvement in mAP. Although FPS decreased by 7, the whole image was within the expected range. After adding the GSConv_C3k2 module to Baseline, Params, and GFLOPs are reduced by 8% and 4.2% while mAP remains unchanged, mainly because the GSConv_C3k2 module combines SC and DSC, and mixes the features generated by standard convolution with those generated by deep convolution through the shuffle, thus enhancing the connection between features and reducing redundant information and unnecessary calculation. By adding the DySample module, Params are reduced by 8% and GFLOPs are reduced by 1.6% because DySample initially employs bilinear interpolation to upsample the input feature map into a continuous representation. Subsequently, it generates content-aware sampling points and resamples this continuous map. This method reduces the number of parameters and computation and significantly reduces the reasoning delay and memory occupation. After adding the SDI module, Params are reduced by 8%, and GFLOPs are reduced by 4.2% because feature fusion has always been the key link to improve model performance in the segmentation task of deep learning. SDI effectively solves the problem of information loss or insufficient fusion through multi-level feature interaction and fusion. SDI makes use of dense connection and phased interaction mechanisms so that different chromatographic features can communicate information more efficiently. After adding the PIoU loss function, mAP decreased by 0.5% and FPS increased by 12.8% because the PIoU loss function guided the anchor frame to regress along a more direct path by combining penalty factor adaptive to target size and gradient adjustment function based on anchor frame quality, thus fitting data more accurately and further optimizing bounding box regression process. And PIOU can better adapt to high aspect ratio targets (cigarettes, eyes closed, etc.).

These results show that the introduction of a single module or loss function in the task of fatigue driving detection can not effectively improve the detection accuracy and model performance. This shows that only relying on a single improvement is not enough to optimize the model, and it is necessary to comprehensively consider various factors to achieve double improvement of detection accuracy and performance.

**Fig 9:**
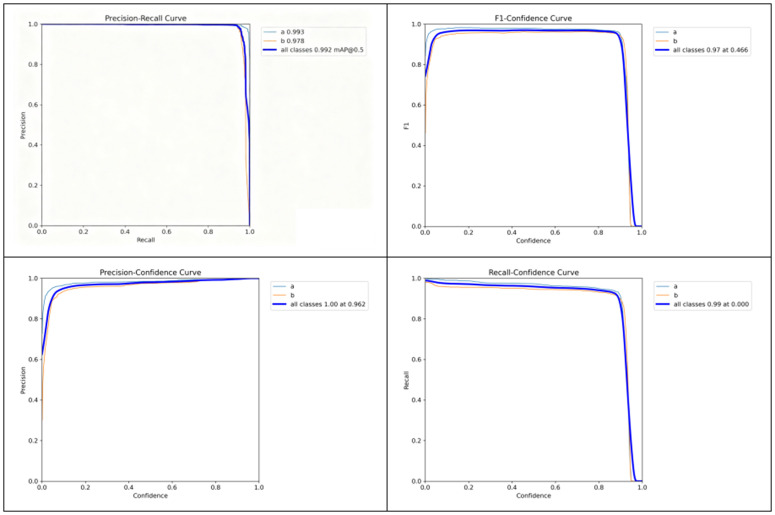
Model state evaluation on the YawDD dataset.

Model State Evaluation on the YawDD Dataset in Fig 9.

Next, we permutate and combine five modules: MSR-LIENET, GSConv_C3k2, DySample, SDI, and PIoU. There is no mutual influence between each module, and each module has its advantages. As detailed in Table 4, each module demonstrates significant independence and effectively contributes to distinct detection stages. Through system-level integration and optimization, the network exhibits enhanced adaptability to complex scenes, collectively improving the accuracy and performance of fatigue-driving object detection. After introducing five modules at the same time, the final model LDIE-FDNet has improved mAP by 0.6%, Params reduce by 24%, GFLOPs improve by 14.3%, and FPS improve by 24.1%. There is no conflict among all modules. Integrating all proposed methods yields the model’s optimal performance.

**Fig 10:**
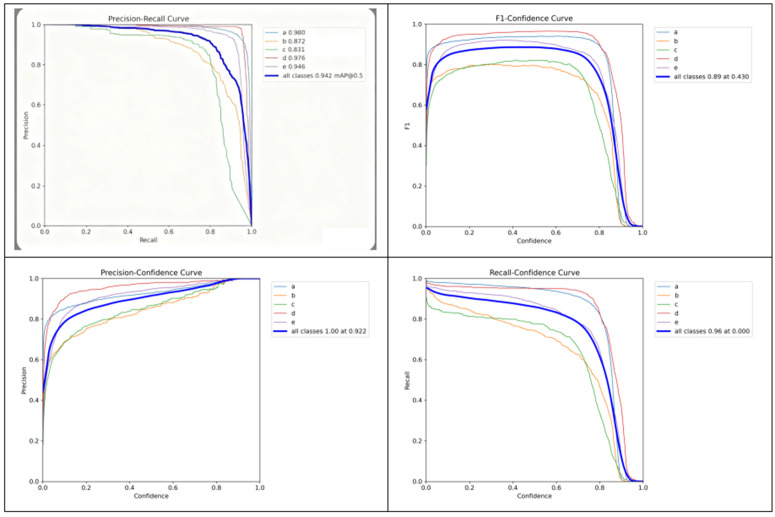
Model state evaluation on the DMS dataset.

Model State Evaluation on the DMS Dataset in Fig 10.

Similar experimental outcomes are observed on the DMS dataset, as detailed in Table 5. Compared to employing a single module, the network integrating stacked blocks demonstrates superior performance. Furthermore, the experiments confirm the compatibility of the proposed modules, with their combined adoption yielding the model’s optimal results.

**Table 5:** Ablation experiment of DMS data set.

MSR-LIENET	GSConv_C3k2	DySample	SDI	PIoU	mAP50(%)	Params(M)	GFLOPs	FPS
×	×	×	×	×	92.1	2.5	6.3	39
√	×	×	×	×	94.2	2.5	6.3	32
×	√	×	×	×	92.0	2.3	5.9	41
×	×	√	×	×	91.9	2.3	6.1	40
×	×	×	√	×	92.2	2.2	6.0	41
×	×	×	×	√	92.2	2.4	6.1	44
√	√	×	×	×	92.9	2.3	5.9	33
√	×	√	×	×	94.1	2.3	6.1	34
√	×	×	√	×	94.0	2.2	6.0	33
√	×	×	×	√	94.0	2.5	6.3	34
×	√	√	×	×	91.9	2.2	5.8	42
×	√	×	√	×	91.9	2.3	6.0	41
×	√	×	×	√	92.1	2.1	5.9	43
×	×	√	√	×	92.0	2.2	6.2	44
×	×	√	×	√	91.7	2.3	6.1	42
×	×	×	√	√	91.9	2.1	6.1	42
√	√	√	×	×	92.9	2.2	5.8	36
√	√	×	√	×	92.8	2.3	6.0	35
√	√	×	×	√	94.0	2.1	5.9	35
√	×	√	√	×	94.0	2.2	6.2	37
√	×	√	×	√	92.9	2.3	6.1	36
√	×	×	√	√	94.0	2.1	6.1	34
×	√	√	√	×	91.6	2.1	5.7	49
×	√	√	×	√	91.7	2.2	5.8	49
×	√	×	√	√	91.6	2.1	5.7	50
×	×	√	√	√	91.6	2.0	5.6	49
√	√	√	√	×	92.8	2.1	5.7	46
√	√	√	×	√	92.9	2.2	5.8	47
√	√	×	√	√	92.8	2.1	5.7	46
√	×	√	√	√	92.8	2.0	5.6	47
×	√	√	√	√	91.5	1.9	5.4	52
√	√	√	√	√	92.7	1.9	5.4	47

#### Comparison without a loss function.

To verify the performance improvement of PIoU loss function in fatigue driving detection task, CIou (Complete Intersection over Union), EIou [38] (Efficient Intersection over Union), SIoU [39] (Soft Intersection over Union) and Shape-IoU [40] (Shape-based Intersection over Union) are used as loss functions of YOLOv11n model respectively and are trained on YawDD data set and compared. Table 6 presents the results.

**Table 6:** Comparison of different loss functions.

Model	mAP50	Params	GFLOPs	FPS
CIoU	98.6%	2.5M	6.3	39
EIoU	98.7%	2.5M	6.4	39
SIoU	98.8%	2.5M	6.6	40
Shape-IoU	98.7%	2.6M	6.7	38
PIoU	98.7%	2.4M	6.2	44

For the YawDD dataset, PIoU delivers competitive mAP50 (0.1% lower than SIoU) alongside notable efficiency gains: 4% more Params, 6.01% higher GFLOPs, and 10% increased FPS. The superior performance arises from PIoUv2’s dual mechanisms: an object-size-adaptive penalty and anchor-quality-based gradient tuning. These features enhance PIoU’s sensitivity to elongated targets (cigarettes, closed eyes), guiding anchors along optimal regression trajectories. Consequently, model convergence speed and regression accuracy improve, rendering PIoUv2 particularly advantageous for latency-sensitive fatigue driving analysis.

#### Qualitative results.

**Fig 11:**
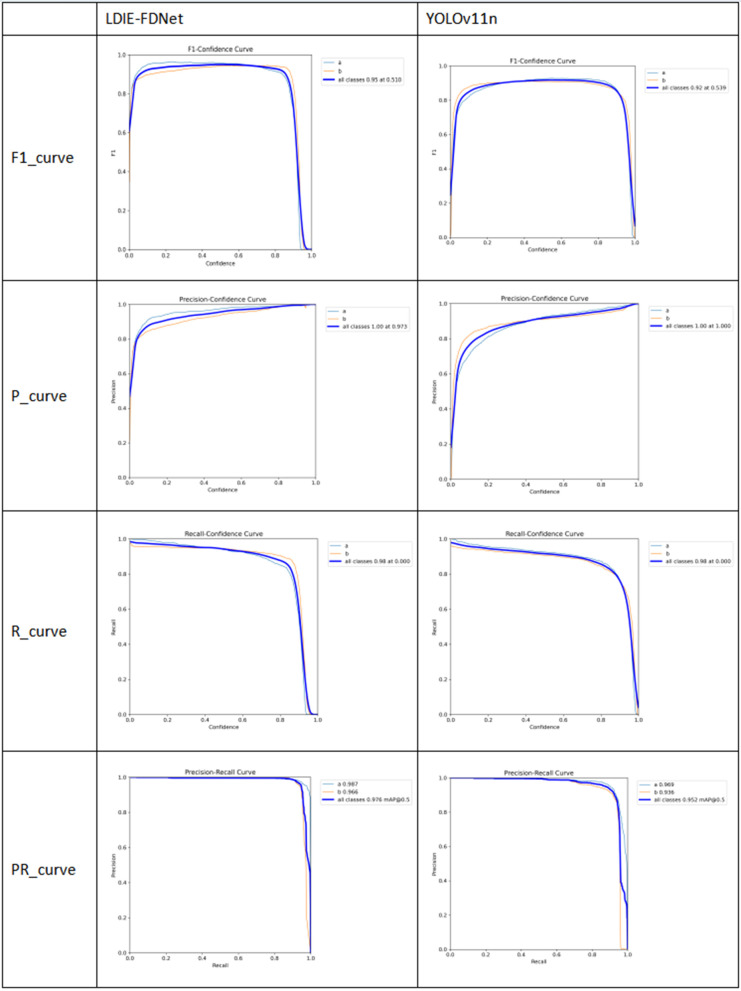
Actual improvement effect in complex driving scenarios.

To validate the practical improvements of MSR-LIENET, DHFAR-Net, and PIoU in complex driving scenarios, we specifically constructed a test dataset containing 500 images with low-light conditions, facial occlusion, and challenging poses. This dataset simulates typical challenging scenarios such as night driving, drivers wearing prescription glasses (instead of sunglasses), and significant head rotations. As shown in Fig 11, we selected representative samples for comparative analysis.

In low-light scenarios, MSR-LIENET significantly enhanced image contrast and detail visibility, transforming previously blurred facial features into clear, distinguishable structures. Compared to the original images, the contours of eyes and mouths became more distinct in MSR-LIENET-processed images, providing high-quality input for subsequent fatigue detection. DHFAR-Net effectively handled complex background interference through dynamic feature aggregation, preventing misidentification of occlusion objects as fatigue behaviors. The PIoU loss function notably improved bounding box regression accuracy, particularly for high-aspect-ratio targets (e.g., closed eyes), achieving better alignment between predicted and ground-truth boxes and reducing false positives and false negatives.

For occlusion scenarios involving prescription glasses, MSR-LIENET improved facial feature visibility by optimizing illumination conditions. DHFAR-Net captured partially visible facial regions through multi-level feature fusion, while PIoU avoided detection box shifts caused by occlusion through precise regression. In challenging pose scenarios with significant head rotations, DHFAR-Net’s dynamic feature aggregation ensured accurate fatigue detection even under non-frontal viewing angles. Meanwhile, PIoU effectively handled high-aspect-ratio targets caused by tilted perspectives, producing detection boxes that better matched actual object shapes.

On this specialized test dataset, our method achieved an mAP50 improvement from 0.952 to 0.976, significantly outperforming baseline models. Specifically, MSR-LIENET improved mAP50 by 2.3% in low-light scenarios, DHFAR-Net enhanced performance by 1.8% in occlusion scenarios, and PIoU contributed a 1.5% mAP50 increase for high-aspect-ratio target detection. These results comprehensively validate the effectiveness of our proposed modules in complex driving environments and provide qualitative visual evidence supporting the claimed improvements.

To address the persistent challenges in extreme occlusion scenarios (e.g., face masks, sunglasses) and motion blur, we conducted a detailed analysis of residual false positives and missed detections. These limitations arise primarily from:

1. Incomplete facial region coverage in masks/sunglasses, reducing key-point visibility;
2. Dynamic noise amplification in motion blur, disrupting temporal feature consistency;
3. Lack of occlusion-aware attention to prioritize visible facial regions.

Future improvements will focus on three directions:

1. Enhanced Face ROI Extraction: Introduce a partial-face detection head that identifies unoccluded regions (e.g., forehead/eyebrows) via keypoint regression, bypassing masked zones.
2. Temporal Smoothing with Motion Compensation: Implement a motion-aware LSTM module to fuse 5 consecutive frames, suppressing blur-induced artifacts through optical flow-guided feature alignment.
3. Occlusion-Aware Multi-Attention: Develop a dual-branch attention mechanism that:Branch A: Focuses on visible facial patches (e.g., eyes under masks);Branch B: Suppresses occlusion artifacts (e.g., sunglasses reflections) via spatial attention masks.

Crucially, this framework maintains backward compatibility with MSR-LIENET’s illumination enhancement and PIoU’s regression stability, ensuring holistic performance gains.

### Comaprative experts of different lightweight algorithms

#### Data set YawDD experiment results.

To compare the detection performance of LDIE-FDNet with other lightweight algorithms, comparative experiments were conducted on the YawDD dataset. As shown in Table 7, LDIE-FDNet not only achieved improved average precision but also demonstrated significant reductions in parameters, computational complexity, and latency (FPS). Compared to the BiFPN architecture, LDIE-FDNet reduced parameters by 2.1M and computational complexity by 1.7 GFLOPs. Against HGNetV2, it reduced parameters by 2.6M and computation by 2.3 GFLOPs. Relative to SlimNeck, parameter and computation reductions were 3.0M and 2.6 GFLOPs, respectively. When compared with EfficientViT, LDIE-FDNet achieved reductions of 2.1M parameters and 4.0 GFLOPs. Versus YOLOv8n, it reduced parameters by 1.0M and computation by 1.3 GFLOPs. Compared to FD-YOLOv8, parameter and computation savings were 0.7M and 1.2 GFLOPs, respectively. Finally, against YOLOv11, LDIE-FDNet reduced parameters by 0.6M and computation by 0.9 GFLOPs.

**Table 7:** Comparative experiments based on YawDD.

Method	P	R	mAP50	mAP50-90	Params	GFLOPs	FPS
BIFPN [41]	94.8	92.7	98.1	89.5	4.0	7.1	15
HGNetV2 [42]	95.3	94.6	98.0	89.8	4.5	7.7	19
Slimneck [43]	96.1	94.8	98.1	90.6	4.9	8.0	28
EfficientViT [44]	96.2	94.9	97.9	90.1	4.0	9.4	36
YOLOv5n	94.8	92.4	94.8	85.6	2.3	5.7	37
EfficientDet-Lite0	86.9	88.4	89.4	76.7	2.0	4.9	30
YOLOv8n [45]	97.3	95.5	98.3	91.1	2.9	6.7	34
FD-YOLOv8 [46]	97.5	95.8	98.5	91.4	2.6	6.6	37
YOLOv11n	98.0	96.2	98.6	92.6	2.5	6.3	39
Ours	98.6	96.7	99.2	92.9	1.9	5.4	48

Real-world fatigue driving detection demands high real-time performance and operates under typically low cabin illumination. To address this, we introduce MSR-LIENET for low-light image enhancement. Concurrently, GSConv_C3k2, DySample, SDI, and PIoU leverage lightweight module advantages, maximizing computational and parametric efficiency while preserving prediction accuracy, thereby enhancing network speed.

This is mainly due to the introduction of MSR-LIENET to enhance the low illumination image and improve the detection accuracy; Add GSConv_C3k2 module to the Baseline to combine SC and DSC, and mix the features generated by standard convolution with those generated by deep convolution through shuffle, to enhance the connection between features and reduce redundant information and unnecessary calculation; The DySample module is added. First, bilinear interpolation converts the input feature map into a continuous representation. Second, content-aware sampling points are generated to resample this continuous map. This approach reduces parametric and computational complexity while substantially decreasing inference latency and memory footprint; After adding the SDI module, through multi-level feature interaction and fusion, the problem of information loss or insufficient fusion is effectively solved. SDI makes use of dense connection and phased interaction mechanism so that different chromatographic features can exchange information more efficiently; The PIoU loss incorporates a target-size-adaptive penalty factor and an anchor-quality-based gradient adjustment function. This dual mechanism steers anchor regression along optimized pathways, enhancing bounding box fitting accuracy and refining the regression process.

Efficiency validation of LDIE-FDNet was conducted on the Jetson Nano platform using the YawDD dataset, with TensorRT optimization implemented to achieve performance improvements through FP16 and INT8 quantization techniques. The detailed comparison results are summarized in Table 8, which meet the real-time requirements.

**Table 8:** Performance comparison across different hardware platforms on YawDD.

Platform	FPS
GTX 1050 Ti	48
Jetson Nano(TensorRT FP16/INT8)	38

#### Data set DMS experiment results.

Experimental results on the DMS dataset confirm our approach’s efficacy against contemporary methods, with quantitative improvements detailed in Table 9. Consistent performance across both DMS and YawDD datasets demonstrates the method’s cross-dataset robustness.

**Table 9:** Comparative experiment of DMS.

Method	P	R	mAP50	mAP50-90	Params	GFLOPs	FPS
BIFPN	84.2	82.9	88.1	64.5	4.0	7.1	15
HGNetV2	84.6	84.1	88.0	62.8	4.5	7.7	19
Slimneck	86.5	84.8	88.1	64.6	4.9	8.0	28
EfficientViT	87.2	84.6	87.9	64.1	4.0	9.4	36
YOLOv5n	87.8	83.2	88.5	63.2	2.3	5.7	36
EfficientDet-Lite0	80.5	79.6	83.5	56.7	2.0	4.9	29
YOLOv8n	88.7	85.7	91.2	66.3	4.9	6.8	34
FD-YOLOv8	89.6	86.2	91.5	67.5	4.6	6.6	37
YOLOv11n	90.7	86.7	92.1	68.4	2.5	6.3	38
Ours	91.4	87.5	94.2	69.7	2.1	5.7	46

Under the same environment, perform efficiency validation on the DMS dataset, the detailed comparison results are summarized in Table 10, which meet the real-time requirements.

**Table 10:** Performance comparison across different hardware platforms on DMS.

Platform	FPS
GTX 1050 Ti	46
Jetson Nano(TensorRT FP16/INT8)	36

## Conclusion

This work proposes LDIE-FDNet: a lightweight dynamic network for real-time fatigue detection. The architecture first employs MSR-LIENET to enhance input illumination, addressing complex lighting challenges to boost detection accuracy. Subsequently, it detects driver behavioral states through optimized inference. Finally, the MCT index and MYD index are designed to judge whether the driver is in a fatigued driving state. Compared with YOLOV11n, mAP50 of the proposed model increases by 0.6% to 99.2%, Params reduce by 24%, GFLOPs increases by 14.3%, FPS increases by 24.1% to 44; Compared with YOLOV11n, mAP50 increases 0.7% to 92.7% and FPS reaches 48 on DMS data set, which verifies that the proposed model has good real-time performance and accuracy in different driving environments.

During the experiment, it was also found that the background, illumination conditions, and personalized differences of driving action amplitude will cause some interference to the model recognition. In future work, it was also proposed to reduce the interference of driver background by adding an attention mechanism. Future work will implement phased video processing to eliminate irrelevant information: first localizing driver faces via object detection, then extracting fatigue features through deep networks. This hierarchical approach aims to enhance detection accuracy, efficiency, and robustness for operational drivers. Additionally, real-vehicle deployment requires further evaluation of algorithm power consumption, reliability, and security.

## References

[1] Shen G, Wang J, Kong X, Ji Z, Zhu B, Qiu T. Deformation Gated Recurrent Network for Lane-level Abnormal Driving Behavior Recognition. ACM Trans Spatial Algorithms Syst. 2024;10(3):1–26. doi: 10.1145/3635141

[2] Zhou F, Alsaid A, Blommer M, Curry R, Swaminathan R, Kochhar D, et al. Driver fatigue transition prediction in highly automated driving using physiological features. Exp Syst Appl. 2020;147:113204. doi: 10.1016/j.eswa.2020.113204

[3] Zhang YF, Gao XY, Zhu JY, Zheng WL. A novel approach to driving fatigue detection using forehead EOG. In: International IEEE/EMBS Conference on Neural Engineering, 2015. 10.1109/NER.2015.7146721

[4] Borghini G, Vecchiato G, Colosimo A, Wei D, Astolfi L. Assessment of Mental Fatigue During Car Driving by Using High Resolution EEG Activity and Neurophysiologic Indices. Conference proceedings:. Annual International Conference of the IEEE Engineering in Medicine and Biology Society. IEEE Engineering in Medicine and Biology Society. Conference. 2012. 10.1109/EMBC.2012.6347469 23367404

[5] Amidei A, Poli A, Iadarola G, Tramarin F, Pavan P, Spinsante S, et al. Driver drowsiness detection based on variation of skin conductance from wearable device. In: 2022 IEEE International Workshop on Metrology for Automotive (MetroAutomotive). 2022. p. 94–8.

[6] Li Z, Chen L, Nie L, Yang SX. A Novel Learning Model of Driver Fatigue Features Representation for Steering Wheel Angle. IEEE Trans Veh Technol. 2022;71(1):269–81. doi: 10.1109/tvt.2021.3130152

[7] Li Z, Li SE, Li R, Cheng B, Shi J. Online Detection of Driver Fatigue Using Steering Wheel Angles for Real Driving Conditions. Sensors (Basel). 2017;17(3):495. doi: 10.3390/s17030495 28257094 PMC5375781

[8] Li Z, Yang Q, Chen S, Zhou W, Chen L, Song L. A fuzzy recurrent neural network for driver fatigue detection based on steering-wheel angle sensor data. Int J Distrib Sensor Netw. 2019;15(9):155014771987245. doi: 10.1177/1550147719872452

[9] McDonald A D, Schwarz C, Lee J D, Brown T L. Real-time detection of drowsiness related lane departures using steering wheel angle. Proceedings of the human factors and ergonomics society annual meeting. Sage CA: Los Angeles, CA: Sage Publications. 2012. 2201–5.

[10] Xu J, Min J, Hu J. Real-time eye tracking for the assessment of driver fatigue. Healthc Technol Lett. 2018;5(2):54–8. doi: 10.1049/htl.2017.0020 29750113 PMC5933402

[11] Knapik M, Cyganek B. Driver’s fatigue recognition based on yawn detection in thermal images. Neurocomputing. 2019;338:274–92. doi: 10.1016/j.neucom.2019.02.014

[12] Sigari MH, Fathy M, Soryani M. A Driver Face Monitoring System for Fatigue and Distraction Detection. Int J Veh Technol. 2013;2013(5):73–100. doi: 10.1155/2013/263983

[13] Zhuang Q, Kehua Z, Wang J, Chen Q. Driver Fatigue Detection Method Based on Eye States With Pupil and Iris Segmentation. IEEE Access. 2020;8:173440–9. doi: 10.1109/ACCESS.2020.3025818

[14] Zhao T, Wang Z, Bai X. Research on fatigue driving detection based on computer vision. 2022 International Conference on Intelligent Transportation, Big Data & Smart City (ICITBS). IEEE; 2022. p. 318–21.

[15] Zhang B. Design of face recognition fatigue driving detection system based on improved YOLO algorithm. In: 2023 International Conference on Mechatronics, IoT and Industrial Informatics (ICMIII), 2023. 602–6.

[16] Xiang W, Wu X, Li C, Zhang W, Li F. Driving Fatigue Detection Based on the Combination of Multi-Branch 3D-CNN and Attention Mechanism. Appl Sci. 2022;12(9):4689. doi: 10.3390/app12094689

[17] Zheng H, Wang Y, Liu X. Adaptive Driver Face Feature Fatigue Detection Algorithm Research. Appl Sci. 2023;13(8):5074. doi: 10.3390/app13085074

[18] Fang Z, Wang J, Wang Z. A human-machine shared control framework considering time-varying driver characteristics. IEEE Trans Intell Veh. 2023;8(7):3826–38. doi: 10.1109/TIV.2023.3268070

[19] Fang Z, Wang J, Liang J, Yan Y, Pi D, Zhang H, et al. Authority Allocation Strategy for Shared Steering Control Considering Human-Machine Mutual Trust Level. IEEE Trans Intell Veh. 2024;9(1):2002–15. doi: 10.1109/tiv.2023.3300152

[20] Selvan PS, Addula SR, Singh CE. Deep learning-enabled fetal health classification through sensor-fused IoT environment. In: 5th International Conference on Mobile Radio Communications and 5G Networks, MRCN 2024. 2025. p. 157–69.

[21] Ji P, Lv Z, Zhang Z. A retinex-based network for low-light image enhancement with multi-scale denoising and focal-aware reflections. IET Image Process. 2025;19(1). doi: 10.1049/ipr2.70059

[22] Wang J, Qin C, Hou B, Yuan Y, Zhang Y, Feng W. LCGSC-YOLO: a lightweight apple leaf diseases detection method based on LCNet and GSConv module under YOLO framework. Front Plant Sci. 2024;15:1398277. doi: 10.3389/fpls.2024.1398277 39544536 PMC11560749

[23] Lin Z, Yun B, Zheng Y. LD-YOLO: A Lightweight Dynamic Forest Fire and Smoke Detection Model with Dysample and Spatial Context Awareness Module. Forests. 2024;15(9):1630. doi: 10.3390/f15091630

[24] Peng Y, Chen D Z, Sonka M. U-net v2: Rethinking the skip connections of u-net for medical image segmentation. 2025 IEEE 22nd International Symposium on Biomedical Imaging (ISBI). IEEE; 2025. p. 1–5.

[25] Chen Z, Chen K, Lin W, See J, Yang C. P I o U Loss: Towards Accurate Oriented Object Detection in Complex Environments. 2020. doi: 10.1007/978-3-030-58558-7_12

[26] Liu Z, Wei L, Song T. Optimized YOLOv11 model for lung nodule detection. Biomed Signal Process Cont. 2025;107:107830. doi: 10.1016/j.bspc.2025.107830

[27] Dong C, Liu J, Xie S. DMSA-Net: a deformable multiscale adaptive classroom behavior recognition network. PeerJ Comput Sci. 2025;11:e2876. doi: 10.7717/peerj-cs.2876 40567793 PMC12192764

[28] Zhang P, Liu X, Yuan J, Liu C. YOLO5-spear: A robust and real-time spear tips locator by improving image augmentation and lightweight network for selective harvesting robot of white asparagus. Biosyst Eng. 2022;218:43–61. doi: 10.1016/j.biosystemseng.2022.04.006

[29] Wang J, Chen K, Xu R, Liu Z, Loy CC, Lin D. CARAFE: Unified Content-Aware ReAssembly of FEatures. IEEE Trans Softw Eng. 2021. doi: 10.1109/TPAMI.2021.3074370 33881989

[30] Do M, Kambhampati S. SAPA: A Multi-objective Metric Temporal Planner. JAIR. 2003;20:155–94. doi: 10.1613/jair.1156

[31] Horn RA. The Hadamard product. Proc Sympos Appl Math. 1990. doi: 10.1090/psapm/040/1059485

[32] Su J, Wang F, Zhuang W. An Improved YOLOv7 Tiny Algorithm for Vehicle and Pedestrian Detection with Occlusion in Autonomous Driving. Chinese J Elect. 2025;34(1):282–94. doi: 10.23919/cje.2023.00.256

[33] Zhou M. LFIR-YOLO: Lightweight Model for Infrared Vehicle and Pedestrian Detection. Sensors. 2024;24. doi: 10.3390/s24206609 PMC11511348 39460089

[34] Mahdi W, Akrout B, Alroobaea R, et al. Automated Drowsiness Detection Through Facial Features Analysis. Computacion y Sistemas. 2019;23(2):511–21. doi: 10.13053/CyS-23-2-3013

[35] Makhmudov F, Turimov D, Xamidov M, Nazarov F, Cho Y-I. Real-Time Fatigue Detection Algorithms Using Machine Learning for Yawning and Eye State. Sensors (Basel). 2024;24(23):7810. doi: 10.3390/s24237810 39686347 PMC11644966

[36] Abtahi S, Omidyeganeh M, Shirmohammadi S, Hariri B. A yawning detection dataset. ACM. 2014. doi: 10.1145/2557642.2563678

[37] Caas PN, Diez A, Galva D, Nieto M, Rodríguez Igor. Occlusion-aware driver monitoring system using the driver monitoring dataset. 2025.

[38] Zhou Q, Liu D, An K. ESE-YOLOv8: A novel object detection algorithm for safety belt detection during working at heights. Entropy. 2024;26(7). doi: 10.3390/e26070591 PMC11275857 39056953

[39] Ning Z, Wang H, Li S, Xu Z. YOLOv7-RDD: A Lightweight Efficient Pavement Distress Detection Model. IEEE Trans Intell Transp Syst. 2024;(7):25. doi: 10.1109/TITS.2023.3347034

[40] Zhang H, Zhang S. Shape-IoU: More Accurate Metric Considering Bounding Box Shape and Scale. 2023.

[41] Arsoy MV, Uysal L. BiFPN-enhanced SwinDAT-based cherry variety classification with YOLOv8. Scientif Rep. 2025. doi: 10.1038/s41598-025-89624-7 PMC11825733 39948150

[42] Xiao S, Liu J, Pan Z, Wang S, Yang Y, Song Z, et al. LiteYOLO-GHG: a lightweight YOLOv8-based algorithm for transformer bushing fault detection. J Supercomput. 2025;81(2). doi: 10.1007/s11227-024-06852-w

[43] Ye Y, Tan G, Liu Q, Liu L, Chu J, Wen B, et al. TSSSKD-YOLO: an intelligent classification and defect detection method of insulators on transmission lines by fusing knowledge distillation in multiple scenarios. Multimed Syst. 2025;31(3). doi: 10.1007/s00530-025-01772-y

[44] Gourisaria MK, Patel AV, Chatterjee R, Singh V. Efficient ViT: An Efficient Vision Transformer for Fire and Smoke Image Classification. In: International Conference on Smart Systems: Innovations in Computing. Springer, Singapore. 2024. 10.1007/978-981-97-3690-4_19

[45] Wan M, Yang X, Zhang H. Waste drilling fluid flocculation identification method based on improved YOLOv8n. Rev Sci Instrum. 2025;96(1):015104. doi: 10.1063/5.0235362 39774912

[46] Wei Z, Liu X, Qiu Y, et al. Fire and Smoke Detection Method in Diverse Scenarios Based on YOLOv8-FD. 2024 5th International Conference on Artificial Intelligence and Computer Engineering (ICAICE).0[2025-06-19]. 10.1109/ICAICE63571.2024.10863950

